# Analysis of OSCE-based clinical competency assessment among critical care medicine residents in Zhejiang Province, China: a multicenter retrospective cohort study

**DOI:** 10.1186/s12909-026-09536-6

**Published:** 2026-05-25

**Authors:** Changqin Chen, Wenchao Mao, Kailun Cai, Caibao Hu, Changyun Zhao

**Affiliations:** Department of Critical Care Medicine, Zhejiang Hospital, Lingyin Road 12, Hangzhou, 310013 Zhejiang China

**Keywords:** Objective Structured Clinical Examination (OSCE), Critical care medicine, Residency training, Clinical competence, Medical education, Clinical reasoning

## Abstract

**Background:**

The Objective Structured Clinical Examination (OSCE) is a standard tool for assessing clinical competence in standardized residency training. However, detailed multi‑station performance analyses focusing on critical care residents are limited. This multicenter retrospective cohort study evaluated OSCE scores across training bases in Zhejiang Province to identify educational gaps and inform training optimization.

**Methods:**

We retrospectively collected clinical competency assessment scores of 175 residents from seven standardized residency training bases (2023–2025), including all eligible residents during the study period (total population sampling; no formal sample size calculation was required). Data were collected using standardized OSCE scoring sheets. The OSCE included seven stations: History Taking (HT), Physical Examination (PE), Initial Medical Progress Note (IMPN), Comprehensive Medical Record (CMR), Clinical Reasoning and Decision‑making (CRDM), Basic Clinical Skills (BCS), and Specialty‑specific Clinical Skills (SCS). Kruskal–Wallis, Mann–Whitney U tests, and Spearman’s correlation with Bonferroni correction were used for analysis.

**Results:**

The overall pass rate was 97.71% (171/175). Significant inter‑base differences were found only for IMPN (*P* = 0.004) and CMR (*P* < 0.001). No significant differences were observed across trainee types or genders. CRDM had the lowest median score (85.0 pts) and widest distribution. Only HT and PE showed a moderate positive correlation (ρ = 0.60, *P* < 0.001 after correction). Annual trends revealed progressive improvement in HT and PE (2025 vs. 2023, both *P* ≤ 0.004), while IMPN and SCS peaked in 2024 then declined (both *P* < 0.001).These results directly address the study aim of comparing performance across bases, trainee types, genders, and years.

**Conclusions:**

Critical care residents performed well in procedural skills, but clinical reasoning remains a notable weakness, and documentation quality varies across bases. These findings support the need for systematic clinical reasoning training, cross-institutional standardization of medical writing, and continuous quality monitoring to improve competency-based education in critical care medicine.

## Introduction

In recent years, China has undergone a profound transformation in its medical education system, most notably with the nationwide implementation of the Standardized Residency Training (SRT) program in 2014 [1, 2]. This reform marked a critical shift from traditional time-based training toward Competency-Based Medical Education (CBME), aiming to homogenize the clinical capabilities of junior physicians across the country and ensure high-quality patient care [3, 4]. Within this framework, critical care medicine represents a particularly demanding specialty. Residents in the intensive care unit (ICU) are required to rapidly master complex life-saving procedures, integrate multidisciplinary medical knowledge, and make critical decisions under extreme pressure [5]. Consequently, the training and assessment of critical care residents demand highly rigorous and objective evaluation methods [6].

The Objective Structured Clinical Examination (OSCE), first introduced by Harden et al. in 1975, has become the internationally recognized gold standard for assessing clinical competence [7, 8]. By utilizing standardized patients (SPs) and advanced medical simulators across multiple timed stations, the OSCE provides a comprehensive and objective evaluation of various clinical domains, including history taking, physical examination, clinical reasoning, and procedural skills [9]. In the context of postgraduate medical education, summative OSCEs serve as a crucial gatekeeping mechanism to ensure that graduating residents possess the essential competencies required for independent clinical practice [10, 11].

Despite the widespread adoption of the SRT program and OSCE assessments in China, variations in training quality and assessment outcomes among different training bases remain a significant concern. Furthermore, there is a scarcity of detailed, multi-station performance analyses specifically focusing on critical care medicine residents. To address this gap, this retrospective study analyzes the final OSCE scores of 175 critical care medicine residents from seven training bases in Zhejiang Province in 2023–2025. By comparing assessment scores across different training bases, trainee types, genders, and years, this study aims to evaluate the current state of critical care residency training, identify specific educational weaknesses, and provide an objective basis for optimizing training pathways.

## Methods

### Study subjects

This multicenter retrospective cohort study collected the final clinical practical skills assessment scores of 175 residents (2023: *n* = 51; 2024: *n* = 71; 2025: *n* = 53) from seven critical care medicine residency training bases (i.e., teaching hospitals certified by the provincial health commission to deliver the standardized residency curriculum in critical care medicine) in Zhejiang Province across three consecutive examination cycles (2023–2025). The residents represented the entire target population (total population sampling) because all eligible trainees from the seven bases during the study period were consecutively included; therefore, no formal sample size calculation was required. Demographic characteristics, including gender, trainee type (unit‑commissioned trainees [UC]: employed by and contracted to return to their sponsoring hospital; professional degree postgraduates [PDP]: enrolled in a concurrent master’s degree program; social trainees [ST]: entered training independently without a prior employment contract), and affiliated training base, were also retrospectively retrieved. All data were derived from official examination records and were fully anonymized prior to analysis by removing resident names and identification numbers and replacing them with a sequential study ID; the mapping file was kept separately and accessible only to a data manager not involved in the analysis. As this study involved non-interventional analysis of de-identified educational assessment data from residents rather than patients, formal ethical review was not required in accordance with applicable institutional regulations.

### OSCE structure and scoring

In accordance with the Regulations for the Final Clinical Practical Skills Assessment of Residency Training (Critical Care Medicine), the final assessment was conducted using the OSCE format. Candidates were evaluated through practical operations on SPs or medical simulators.

The OSCE consisted of seven independent stations: History Taking (HT), Physical Examination (PE), Initial Medical Progress Note (IMPN), Comprehensive Medical Record (CMR), Clinical Reasoning and Decision-making (CRDM), Basic Clinical Skills (BCS; including cardiopulmonary resuscitation and endotracheal intubation), and Specialty-specific Clinical Skills (SCS).

The maximum score for the Specialty-specific Clinical Skills station was 110 points and was converted to a 100-point scale for comparability; all other stations had a maximum score of 100 points. The passing threshold for every individual station was set at 80 points. This threshold follows the provincial standard mandated by the Zhejiang Provincial Health Commission for the final clinical practical skills assessment of residency training [12]. At the provincial level, all stations are assessed using centrally calibrated scoring rubrics, and examiners receive standardized training prior to each examination cycle to ensure comparability across stations. No additional standard‑setting study (e.g., modified Angoff or borderline regression method) was performed for this retrospective analysis, as the passing mark was predetermined by the examination regulations [13]. A candidate was required to pass all stations to achieve an overall passing grade. During the examination two examiners (attending physicians or above in critical care medicine, all with prior OSCE examiner training) independently evaluated each candidate simultaneously, and the average of their scores was recorded as the final score for that station. Data were collected using standardized OSCE scoring sheets. This study comparatively analyzed assessment scores across training bases, trainee types, genders, and examination years (2023–2025) to provide an objective basis for optimizing the training pathways of critical care medicine residents.

### Statistical analysis

Statistical analyses were performed using SPSS version 25.0 (IBM Corp., Armonk, NY, USA) and Python 3.11. The normality of all continuous variables was assessed using the Shapiro-Wilk test. To determine whether to use parametric or non‑parametric methods for between‑base comparisons, we applied the following prespecified rule: for each OSCE station, we tested score normality separately within each training base (α = 0.05; bases with n < 3 were excluded). If any base violated normality for a given station, that station was analysed non‑parametrically using the Kruskal‑Wallis H test and reported as median (Q1, Q3). Only if all tested bases passed normality for a station did we use one‑way ANOVA with mean ± SD. Non-normally distributed data are expressed as median with interquartile range [M (Q1, Q3)]. The Mann-Whitney U test was used for comparisons between two independent groups. The Kruskal-Wallis H test was used for comparisons among multiple groups, with Bonferroni correction applied for pairwise post-hoc comparisons. This approach was applied to comparisons across training bases, trainee types, and examination years (2023, 2024, and 2025); for year-wise pairwise comparisons, the corrected threshold was α’ = 0.05/3 ≈ 0.0167. Inter-station correlations were assessed using Spearman’s rank correlation, with Bonferroni correction across all 21 pairwise comparisons (α’ = 0.05/21 ≈ 0.0024). Data visualization was performed using the matplotlib and seaborn libraries in Python 3.11. All tests were two-tailed, and *P* < 0.05 was considered statistically significant.

## Results

### Baseline characteristics of residents

A total of 175 residents from 7 critical care medicine residency training bases in Zhejiang Province were enrolled across three consecutive examination cycles (2023: *n* = 51; 2024: *n* = 71; 2025: *n* = 53). The cohort comprised 97 males (55.43%) and 78 females (44.57%). The majority were unit-commissioned trainees (*n* = 120, 68.57%), followed by professional degree postgraduates (*n* = 50, 28.57%) and social trainees (*n* = 5, 2.86%). The overall pass rate was 97.71% (171/175). Detailed score distributions across training bases are presented in Table 1.

### Comparison of assessment results across different training bases

Applying the prespecified normality‑based rule (see Methods), CRDM was the only station for which all seven training bases passed the Shapiro‑Wilk test (minimum *p* = 0.108); therefore, CRDM is reported as mean ± SD with one‑way ANOVA, while all other stations are reported as median (IQR) with Kruskal‑Wallis tests. Table 1 presents OSCE station scores across seven critical care training bases (*N* = 175, 2023–2025). Kruskal‑Wallis tests showed significant inter‑base differences only for the Initial Medical Progress Note (IMPN; H = 19.040, *P* = 0.004) and the Comprehensive Medical Record (CMR; H = 57.690, *P* < 0.001). No significant differences were found for the other six stations (all *P* > 0.05), indicating comparable training quality in clinical skills across bases.

For CMR (overall median: 92.25), Base D scored highest (94.50) and Base A lowest (88.62); post‑hoc comparisons (Bonferroni) revealed significant differences between Base D and Bases A, B, C, E, and F (all *P* < 0.05). For IMPN (overall median: 88.50), Base D performed best (91.88) and Base G lowest (86.25), with a significant difference between Base B and Base D (*P* = 0.022). These findings suggest that while clinical procedural training is well standardized across bases, medical documentation training requires targeted quality improvement.

**Table 1:** Comparison of OSCE station scores across training bases in critical care medicine residency training (2023–2025) [Score, Mean ± SD or M (Q1, Q3)]

Station	Training Base							Overall (*n* = 175)	Statistic	*P* value
	Base A (*n* = 36)	Base B (*n* = 39)	Base C (*n* = 24)	Base D (*n* = 36)	Base E (*n* = 17)	Base F (*n* = 15)	Base G (*n* = 8)			
History Taking (HT)	92.12 (89.94, 94.81)	92.50 (88.62, 94.50)	91.62 (87.12, 93.62)	92.00 (86.19, 94.50)	90.75 (88.25, 95.50)	93.25 (91.12, 94.50)	91.12 (90.12, 91.88)	92.00 (88.25, 94.50)	H = 3.135	0.792
Physical Examination (PE)	93.00 (90.00, 95.38)	92.25 (88.38, 94.62)	92.12 (90.44, 95.81)	91.12 (89.25, 93.44)	93.50 (89.00, 96.75)	93.50 (86.38, 94.25)	91.50 (88.75, 93.69)	92.25 (89.00, 95.00)	H = 3.934	0.686
Initial Medical Progress Note (IMPN)	89.50 (87.06, 92.25)	88.50 (84.62, 90.88)	88.00 (87.19, 90.31)	91.88 (88.44, 95.88)	87.00 (86.25, 89.50)	88.75 (84.62, 94.50)	86.25 (85.25, 87.12)	88.50 (86.00, 91.75)	H = 19.040	0.004*
Comprehensive Medical Record (CMR)	88.62 (86.56, 91.44)	91.75 (89.88, 92.62)	92.75 (91.25, 93.50)	94.50 (93.25, 96.31)	92.25 (90.25, 94.25)	93.75 (91.88, 97.88)	93.12 (91.75, 94.62)	92.25 (90.12, 94.12)	H = 57.690	< 0.001*
Clinical Reasoning & Decision-making (CRDM) ‡	85.78 ± 3.49	84.24 ± 3.66	85.88 ± 4.87	85.48 ± 3.38	85.97 ± 4.07	86.58 ± 5.21	82.53 ± 4.22	85.33 ± 4.01	F = 1.640	0.139
Basic Clinical Skills (BCS)	93.50 (90.75, 95.81)	93.50 (91.50, 95.88)	94.25 (92.50, 95.94)	93.88 (91.69, 94.75)	94.50 (92.25, 95.75)	93.25 (90.38, 94.25)	90.00 (88.94, 95.12)	93.75 (91.12, 95.50)	H = 6.215	0.400
Specialty Clinical Skills (SCS)†	92.16 (87.62, 96.88)	93.64 (87.28, 96.13)	93.18 (87.16, 95.28)	93.75 (91.59, 96.42)	91.82 (87.95, 93.86)	94.55 (92.50, 96.36)	93.06 (86.71, 94.32)	93.18 (89.20, 95.91)	H = 7.860	0.249
Total Score	640.88 (634.31, 652.12)	640.75 (633.62, 647.25)	641.12 (634.94, 651.31)	649.88 (636.62, 661.81)	644.00 (641.75, 649.00)	651.50 (637.12, 657.25)	634.25 (626.62, 643.69)	642.50 (634.75, 653.12)	H = 9.907	0.129

Bold values in the table indicate results with statistically significant between‑group differences

*P < 0.05. † SCS scores were converted from the original 110-point scale to a 100-point scale (×10/11) prior to analysis and presentation. Normally distributed data are expressed as Mean ± SD (one-way ANOVA); non-normally distributed data are expressed as M (Q1, Q3) (Kruskal-Wallis H test). ‡CRDM was the only station that passed normality testing for all seven bases (Shapiro‑Wilk *p* ≥ 0.108 for each base); therefore, CRDM is reported as Mean ± SD with one‑way ANOVA. All other stations had at least one base with *p* < 0.05, thus are reported as median (Q1, Q3) with Kruskal‑Wallis H tests

### Comparison of assessment results among different trainee types

**Fig. 1:**
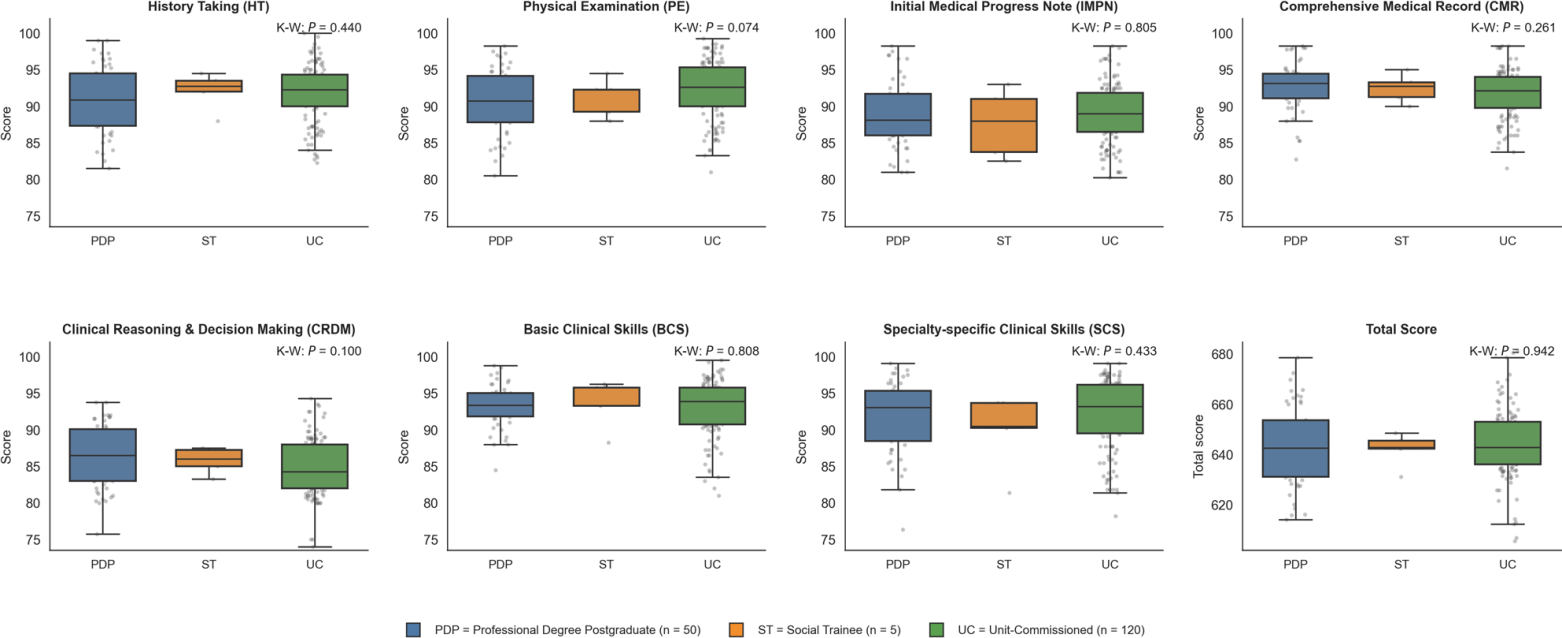
Comparison of OSCE station scores by trainee type

Kruskal-Wallis H tests revealed no significant differences in OSCE scores among Professional Degree Postgraduates (PDP, *n* = 50), Social Trainees (ST, *n* = 5), and Unit-Commissioned trainees (UC, *n* = 120) across all seven stations or total scores (all *P* > 0.05; Fig. 1). Median scores and interquartile ranges were comparable across groups, indicating that the standardized training curriculum effectively equalizes clinical competency outcomes regardless of trainees’ educational backgrounds or employment status.

Box plots show score distributions for PDP (*n* = 50), ST (*n* = 5), and UC (*n* = 120) across seven OSCE stations and total scores. Box boundaries represent the IQR; black lines indicate medians; whiskers extend to 1.5×IQR; dots show individual data points. Kruskal-Wallis H test P values are annotated in each panel. No significant differences were observed (all *P* > 0.05)

### Comparison of assessment results between different genders

**Fig. 2:**
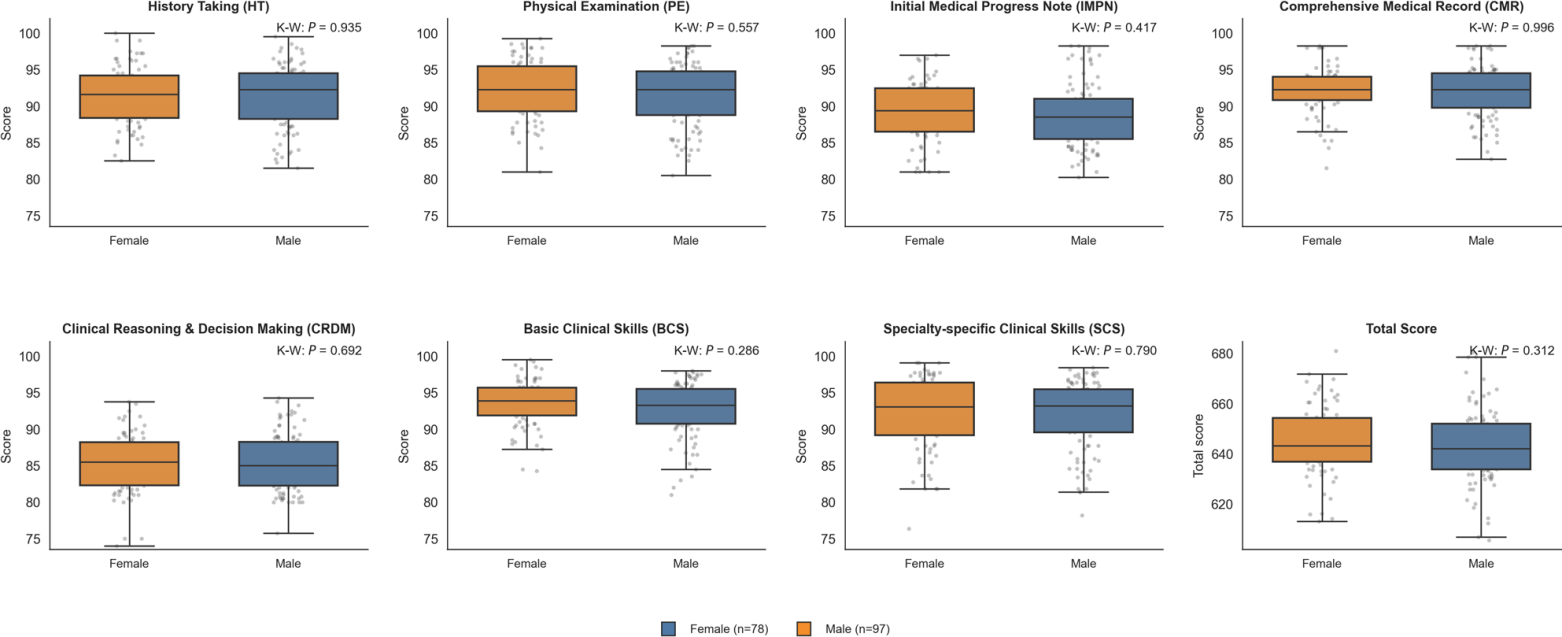
Comparison of OSCE station scores between male and female residents (2023–2025). Box plots display the median (horizontal line), interquartile range (IQR; box boundaries), and 1.5×IQR (whiskers). Grey dots represent individual data points. Group differences were assessed using the Mann-Whitney U test; P values are annotated in the upper right corner of each panel. No statistically significant differences were observed between genders in any station (all *P* > 0.05). Female, *n* = 78; Male, *n* = 97

Of the 175 residents, 97 were male (55.43%) and 78 were female (44.57%). Mann-Whitney U tests revealed no statistically significant differences between male and female residents in any OSCE station or in the total score (all *P* > 0.05; Fig. 2), indicating that gender did not influence clinical competency assessment performance.

### Comparison of OSCE scores across different stations

**Fig. 3:**
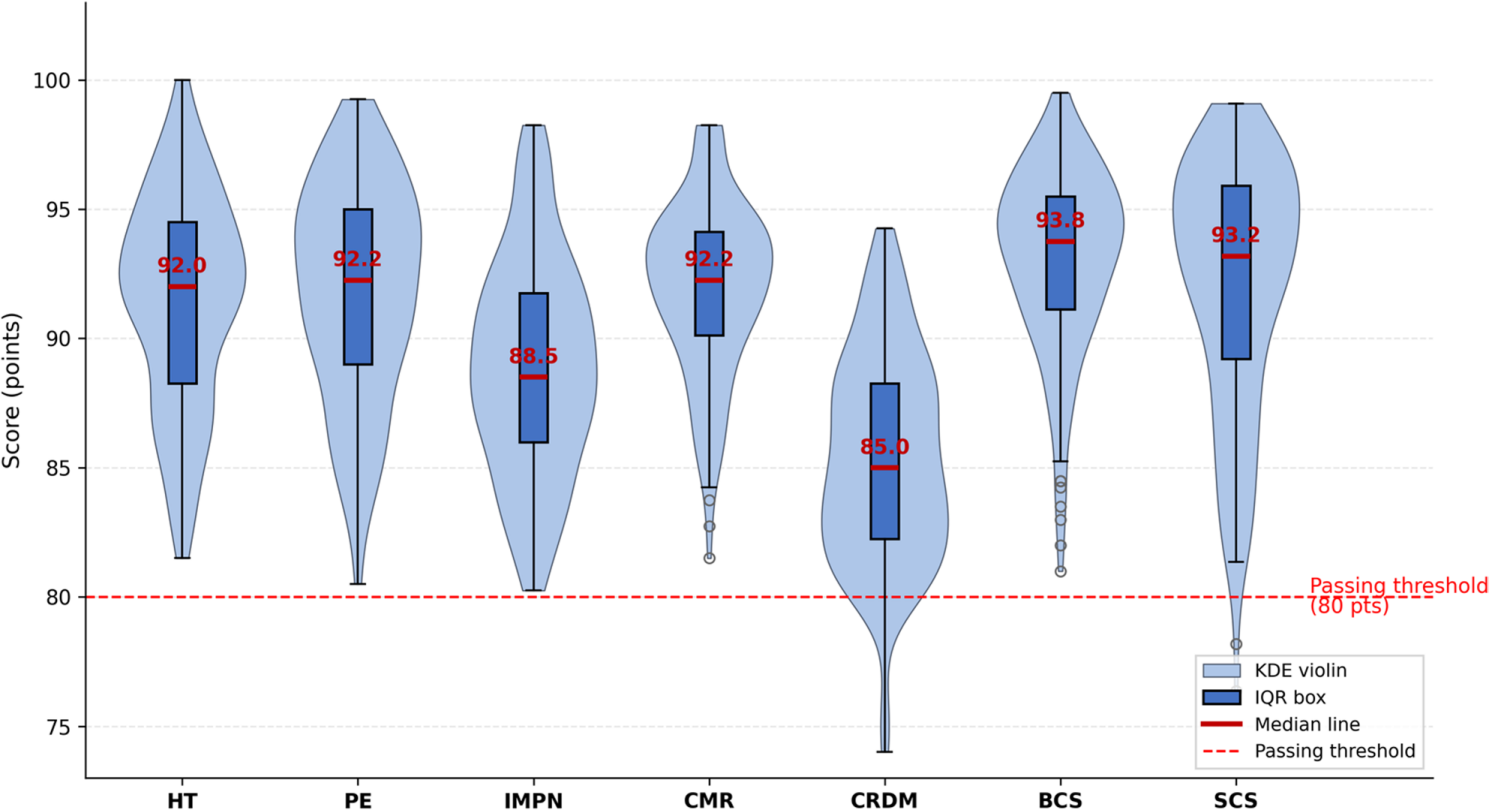
Distribution of OSCE station scores across all residents (*N* = 175, 2023–2025). The shaded area represents the kernel density estimate (KDE) of score distribution. The inner box indicates the interquartile range (IQR; Q1–Q3), the bold red line denotes the median with the value annotated, and whiskers extend to 1.5×IQR. Open circles represent outliers. The red dashed line indicates the passing threshold (80 points). HT, History Taking; PE, Physical Examination; IMPN, Initial Medical Progress Note; CMR, Comprehensive Medical Record; CRDM, Clinical Reasoning & Decision-making; BCS, Basic Clinical Skills; SCS, Specialty-specific Clinical Skills (converted to 100-point scale)

The distribution of scores across the seven OSCE stations is presented in Fig. 3. All stations demonstrated median scores above the passing threshold of 80 points. Basic Clinical Skills (BCS) achieved the highest median score (93.8 pts), followed by Specialty-specific Clinical Skills (SCS; 93.2 pts), History Taking (HT; 92.0 pts), Physical Examination (PE; 92.2 pts), and Comprehensive Medical Record (CMR; 92.2 pts). In contrast, Clinical Reasoning & Decision-making (CRDM) yielded the lowest median score (85.0 pts) and the widest score distribution, with the violin plot extending below the passing threshold, indicating that a small subset of residents scored below 80 points in this station. Initial Medical Progress Note (IMPN) also showed a relatively lower median (88.5 pts) with greater score dispersion compared to the procedural stations.

### Inter-station correlations

**Fig. 4:**
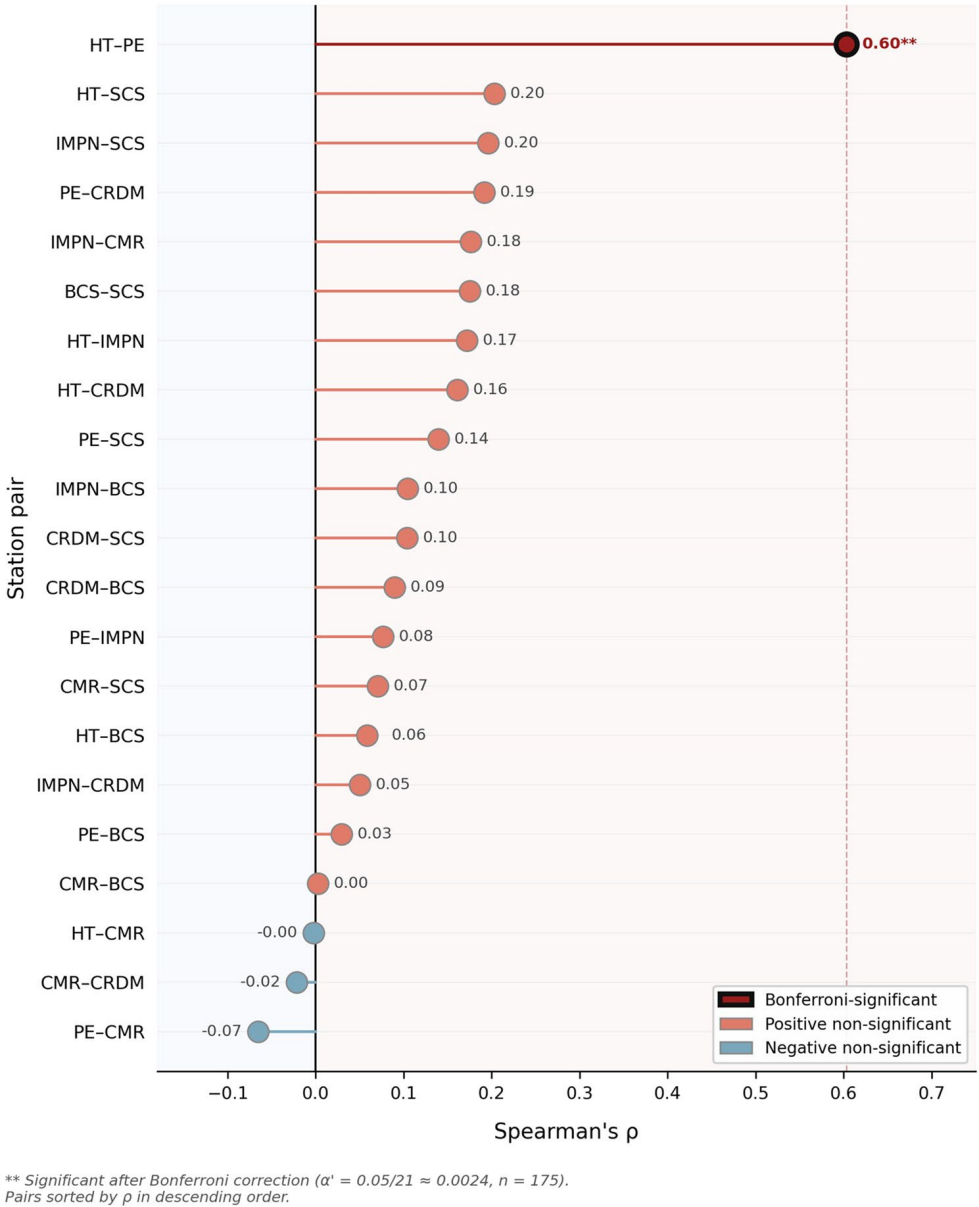
Pairwise spearman correlation coefficients among seven OSCE stations (*N* = 175). All 21 station pairs are sorted by ρ in descending order. The deep-red lollipop indicates the only Bonferroni-significant pair (α’ ≈ 0.0024); ** denotes *P* < 0.001. Coral and blue lollipops represent positive and negative non-significant pairs, respectively. The black vertical line marks ρ = 0; the red dashed line marks ρ = 0.60. HT, History Taking; PE, Physical Examination; IMPN, Initial Medical Progress Note; CMR, Comprehensive Medical Record; CRDM, Clinical Reasoning & Decision-making; BCS, Basic Clinical Skills; SCS, Specialty-specific Clinical Skills

Spearman correlation analysis with Bonferroni correction (21 comparisons; α’ = 0.05/21 ≈ 0.0024) revealed that only the History Taking–Physical Examination pair reached statistical significance (ρ = 0.60, *P* < 0.001; Fig. 4). All remaining 20 pairs showed weak correlations (|ρ| ≤ 0.20, all *P* > 0.0024), including two weakly negative pairs (PE–CMR, ρ = −0.07; CMR–CRDM, ρ = −0.02), indicating that the seven stations largely assessed independent competency domains.

### Annual trends in OSCE station performance (2023–2025)

**Fig. 5:**
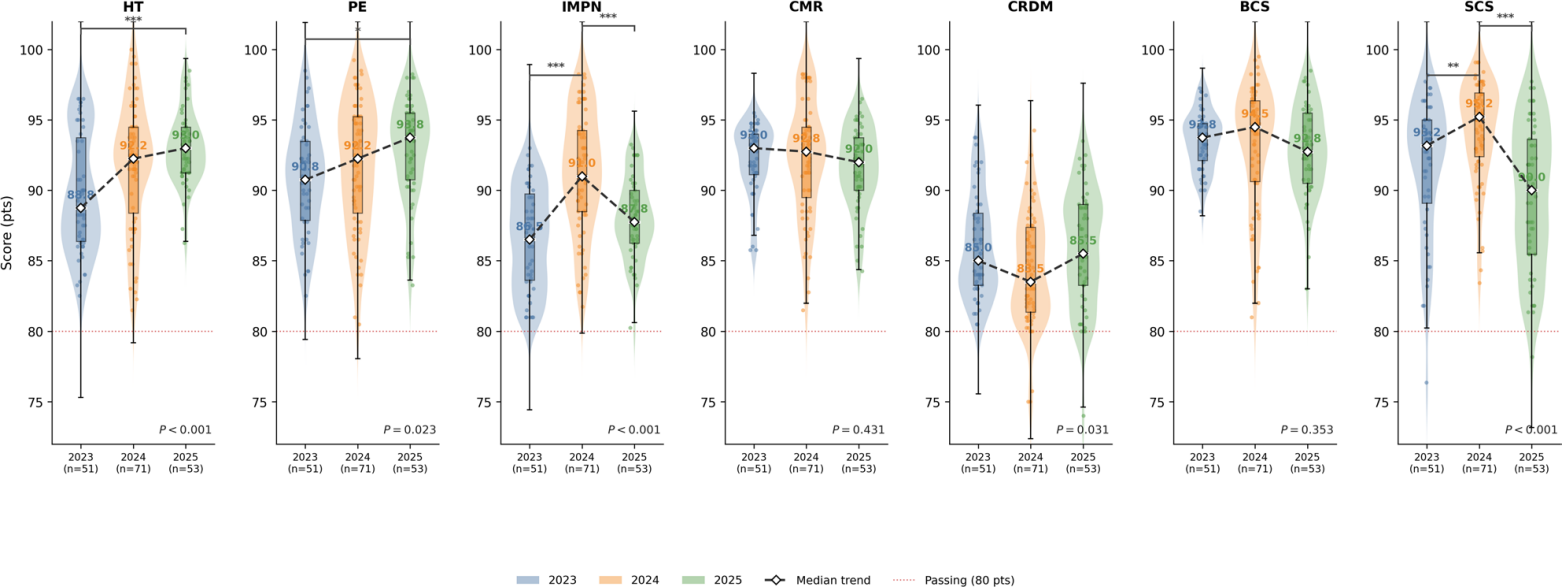
Annual trends in OSCE station scores among critical care medicine residents (2023–2025). Violin plots display score distributions per year (2023, n = 51; 2024, n = 71; 2025, n = 53), overlaid with box plots (IQR; whiskers to 1.5×IQR) and jittered individual data points. Dashed lines connect annual medians (hollow diamonds). The red dotted line indicates the passing threshold (80 pts). Overall inter-annual differences were assessed by the Kruskal-Wallis H test (P values, bottom right). Significant pairwise differences after Bonferroni correction (α’ ≈ 0.0167) are indicated by brackets (** *P* < 0.01, *** *P* < 0.001). HT, History Taking; PE, Physical Examination; IMPN, Initial Medical Progress Note; CMR, Comprehensive Medical Record; CRDM, Clinical Reasoning & Decision-making; BCS, Basic Clinical Skills; SCS, Specialty-specific Clinical Skills

Kruskal-Wallis tests identified significant inter-annual differences in five stations: HT, PE, IMPN, CRDM, and SCS (all P < 0.05), while CMR and BCS remained stable (both P > 0.05; Fig. 5). Post-hoc analyses (Bonferroni, α’ = 0.0167) showed that HT and PE scores improved progressively, with 2025 significantly exceeding 2023 (HT: 93.0 vs. 88.8; PE: 93.8 vs. 90.8; both *P* ≤ 0.004). In contrast, IMPN and SCS peaked in 2024 then declined in 2025 (IMPN: 91.0 vs. 87.8; SCS: 95.2 vs. 90.0; both *P* < 0.001). No pairwise difference survived correction for CRDM.

## Discussion

This multicenter, retrospective study evaluated the clinical competency of 175 critical care medicine residents across seven training bases in Zhejiang Province from 2023 to 2025 using a seven-station OSCE. Our findings demonstrate that while residents achieved high proficiency in basic and specialty-specific clinical skills, significant challenges remain in clinical reasoning and medical documentation. Furthermore, we identified significant inter-institutional variations and dynamic annual trends, providing valuable insights for optimizing competency-based medical education (CBME) in critical care.

Interpreted through Miller’s pyramid of clinical competence, the OSCE assesses the “shows how” level – performance in a simulated, controlled setting [14]. A primary finding of this study is the distinct performance disparity across OSCE stations. BCS and SCS yielded the highest median scores (93.8 and 93.2, respectively) with narrow distributions, indicating that procedural skills are effectively taught and standardized within the current residency curriculum. In stark contrast, the CRDM station recorded the lowest median score (85.0) and the widest dispersion, with a subset of residents failing to meet the passing threshold.

This discrepancy aligns with broader trends in medical education. Clinical reasoning is a complex, multi-component construct that requires the integration of knowledge, pattern recognition, and decision-making under uncertainty—skills that are inherently more difficult to acquire and assess than procedural tasks [15, 16]. Studies have consistently shown that clinical reasoning stations in high-stakes OSCEs yield lower scores and higher variance compared to technical skills [17, 18]. In the high-acuity environment of the intensive care unit (ICU), where decisions must often be made rapidly with incomplete data, the cognitive load is exceptionally high [5]. The wide dispersion in CRDM scores suggests that while current training paradigms excel at imparting technical proficiency, they may fall short in systematically developing high-level cognitive skills. Addressing this gap requires targeted interventions, such as incorporating Script Concordance Tests (SCT) or post-station oral debriefings, which have been shown to effectively stimulate and evaluate clinical reasoning [19, 20]. Furthermore, fostering a clinical practice culture that actively models reasoning behaviors during bedside rounds is crucial for residents’ cognitive maturation [21].

Our correlation analysis revealed that the seven OSCE stations largely function as independent assessment units. Following stringent Bonferroni correction, only the HT and PE stations demonstrated a significant, moderate positive correlation (ρ = 0.60). This specific correlation is clinically intuitive, as patient encounters naturally integrate history gathering and physical assessment. The lack of significant correlation among the remaining 20 station pairs supports the construct validity of the multi-station OSCE design. Previous meta-analyses and multicenter studies have established that low inter-station reliability (often reflected by Cronbach’s alpha values between 0.40 and 0.65) is typical in OSCEs, indicating that clinical competence is not a single unified trait but a composite of distinct domains [22–24]. For instance, a resident’s ability to perform endotracheal intubation (BCS) does not inherently predict their proficiency in synthesizing a CMR or navigating complex diagnostic dilemmas (CRDM). This independence reinforces the necessity of comprehensive, multi-modal assessments to capture the full spectrum of a resident’s capabilities [25, 26].

While procedural skills were comparable across the seven training bases, significant inter-institutional differences emerged in the Initial Medical Progress Note (IMPN) and CMR stations. This finding is consistent with national studies highlighting substantial hospital-level variation in learner milestone ratings and examiner standards [27, 28]. Medical documentation requires not only medical knowledge but also adherence to specific institutional protocols and writing cultures. The observed disparities suggest that some bases may lack standardized training modules for clinical writing, pointing to a need for cross-institutional calibration and shared grading rubrics [29].

Longitudinal analysis of the 2023–2025 cohorts revealed distinct developmental trajectories across competencies. HT and PE scores demonstrated a progressive, significant improvement over the three years, reflecting successful curriculum adaptations and the cumulative benefit of structured patient-encounter training [30]. Notably, CRDM scores remained consistently low without significant pairwise improvement across the years, underscoring the persistent difficulty of teaching and evaluating clinical reasoning within the standard residency timeframe [31].

The most striking annual finding, however, was the non‑linear pattern of IMPN and SCS scores, which peaked in 2024 and then declined in 2025.Conversely, IMPN and SCS scores exhibited non-linear patterns, peaking in 2024 before declining in 2025. While such fluctuations in complex skill domains have been reported in longitudinal OSCE studies [17], our dataset does not contain detailed information on cohort composition, examiner calibration sessions, or specific training module modifications between 2024 and 2025. Therefore, we cannot empirically distinguish among the potential explanations proposed in the literature (e.g., changes in trainee baseline characteristics, year‑to‑year variability in examiner stringency, or transient disruptions in teaching delivery). Consequently, this finding should be interpreted as hypothesis‑generating. To explain the observed decline, we recommend that future prospective studies or quality improvement initiatives conduct: (i) an annual examiner calibration audit to track inter‑rater consistency over time; (ii) a structured curriculum review, focusing on whether the teaching hours or feedback quality for IMPN and SCS were maintained or altered between 2024 and 2025; and (iii) a retrospective survey of program directors to identify any site‑level disruptions (e.g., staffing changes, simulator availability) that might have affected SCS training. Such investigations would provide actionable insights for stabilising and improving performance in these essential competency domains.

Reassuringly, our analysis found no statistically significant differences in OSCE performance across genders or trainee types (Professional Degree Postgraduates, Social Trainees, and Unit-Commissioned residents). Historically, studies in undergraduate medical education have reported gender differences—such as female students excelling in communication and empathy [32], or male students initially outperforming in surgical simulations [33]. However, literature on graduate medical education indicates that structured, competency-based residency programs with standardized feedback mechanisms effectively neutralize these initial gaps [34, 35]. The parity observed in our study suggests that the SRT curriculum in Zhejiang Province provides equitable educational opportunities and successfully standardizes clinical competency regardless of a resident’s background or gender [36].

This study has several limitations. First, as a retrospective analysis restricted to a single province, the findings may not be generalizable to other regions with different healthcare resources. Second, while the OSCE is a robust assessment tool, it evaluates performance in a simulated environment, and its test construction has inherent limitations: the checklist‑based scoring may favour formulaic performance over deep cognitive processing, and its appropriateness for assessing clinical reasoning has been questioned [15, 20]. Consequently, the low CRDM scores observed in our study could partly reflect insufficient sensitivity of the OSCE to capture authentic reasoning rather than a true lack of competence. Additionally, we did not analyze specific sub‑components within the CRDM station, which could provide more granular insights into cognitive bottlenecks. Third, the uniform 80‑point passing threshold was the provincial standard mandated by the Zhejiang Provincial Health Commission rather than a threshold derived from formal standard‑setting methods (e.g., modified Angoff or borderline regression) tailored to each station [37, 38]. Without such methods, some residents who failed the CRDM station might be false negatives. Future studies should apply station‑specific cut‑score methods and compare OSCE results with workplace‑based assessments to validate interpretations of clinical reasoning deficits. Moreover, the trainee‑type comparison included a Social Trainee subgroup of only five individuals, whereas the other two groups comprised 50 and 120 residents. With such a small sample, the Kruskal‑Wallis test has very low statistical power to detect true differences; therefore, the null result for trainee‑type should be interpreted with caution. Finally, because our retrospective dataset only contained the averaged scores from the two examiners per station (the original independent ratings were not retained), we could not compute inter‑rater reliability statistics (e.g., ICC) for the dual‑examiner scoring process. Prospective OSCE implementations should routinely store independent rater scores to enable such analyses.

## Conclusion

In conclusion, the OSCE is a highly valid and multidimensional tool for assessing critical care medicine residents. Our findings reveal two actionable priorities for program directors and training institutions. First, given that clinical reasoning was the lowest‑scoring domain with the widest variance, we recommend integrating structured cognitive training into routine ICU teaching—for example, weekly script concordance test (SCT) exercises, post‑simulation debriefings focused on diagnostic decision‑making, and bedside reasoning case discussions. Second, the significant inter‑institutional variability in medical documentation (IMPN and CMR) calls for cross‑base standardization: institutions should adopt shared grading rubrics, conduct annual examiner calibration workshops, and implement peer review of written records. Finally, because procedural skills (BCS, SCS) are already well taught, future quality improvement efforts should reallocate training time toward reasoning and documentation without compromising technical proficiency. Moving from ‘what we found’ to ‘what to do differently,’ these recommendations provide a concrete roadmap for competency‑based critical care education.

## Data Availability

The datasets used and/or analysed during the current study are available from the corresponding author on reasonable request.

## Abbreviations

BCS: Basic Clinical Skills
CBME: Competency-Based Medical Education
CMR: Comprehensive Medical Record
CRDM: Clinical Reasoning and Decision-making
DOPS: Direct Observation of Procedural Skills
HT: History Taking
ICU: Intensive Care Unit
IQR: Interquartile Range
IMPN: Initial Medical Progress Note
KDE: Kernel Density Estimate
Mini-CEX: Mini Clinical Evaluation Exercise
OSCE: Objective Structured Clinical Examination
PDP: Professional Degree Postgraduate
PE: Physical Examination
SCT: Script Concordance Test
SCS: Specialty-specific Clinical Skills
SP: Standardized Patient
SRT: Standardized Residency Training
ST: Social Trainee
UC: Unit-Commissioned trainee

## References

[1] Zhu J, Li W, Chen L. Doctors in China: improving quality through modernisation of residency education. Lancet. 2016;388(10054):1922–9. 27339756 10.1016/S0140-6736(16)00582-1

[2] Lio J, et al. Standardized residency training in China: the new internal medicine curriculum. Perspect Med Educ. 2018;7(1):50–3. 29098637 10.1007/s40037-017-0378-5 PMC5807259

[3] Li HC, Wang C. Reform direction of medical education in China: implementing competency-based medical education. Chin Med J (Engl). 2013;126(17):3203–4. 24033936

[4] Fraser AB, Stodel EJ, Chaput AJ. Curriculum reform for residency training: competence, change, and opportunities for leadership. Can J Anaesth. 2016;63(7):875–84. 27044399 10.1007/s12630-016-0637-7

[5] Castellanos-Ortega Á, et al. Competency assessment of residents of Intensive Care Medicine through a simulation-based objective structured clinical evaluation (OSCE). A multicenter observational study. Med Intensiva (Engl Ed). 2022;46(9):491–500. 36057440 10.1016/j.medine.2022.01.001

[6] Li L, et al. Chinese critical care certified course in intensive care unit: a nationwide-based analysis. BMC Med Educ. 2023;23(1):576. 37582757 10.1186/s12909-023-04534-4 PMC10428552

[7] Harden RM, et al. Assessment of clinical competence using objective structured examination. Br Med J. 1975;1(5955):447–51. 1115966 10.1136/bmj.1.5955.447 PMC1672423

[8] Khan KZ, et al. The Objective Structured Clinical Examination (OSCE): AMEE Guide 81. Part I: an historical and theoretical perspective. Med Teach. 2013;35(9):e1437–46. 23968323 10.3109/0142159X.2013.818634

[9] Wallenstein J, et al. A core competency-based objective structured clinical examination (OSCE) can predict future resident performance. Acad Emerg Med. 2010;17(Suppl 2):S67–71. 21199087 10.1111/j.1553-2712.2010.00894.x

[10] Zhang H, et al. Network analysis of an OSCE-based graduation skills assessment for clinical medical students. BMC Med Educ. 2025;25(1):605. 40275224 10.1186/s12909-025-07091-0 PMC12023391

[11] Zayyan M. Objective structured clinical examination: the assessment of choice. Oman Med J. 2011;26(4):219–22. 22043423 10.5001/omj.2011.55 PMC3191703

[12] Yousuf N, Violato C, Zuberi RW. Standard Setting Methods for Pass/Fail Decisions on High-Stakes Objective Structured Clinical Examinations: A Validity Study. Teach Learn Med. 2015;27(3):280–91. 26158330 10.1080/10401334.2015.1044749

[13] Taylor J, et al. Implementation of standard setting for high-stakes objective structured clinical examinations. Curr Pharm Teach Learn. 2024;16(6):465–8. 38582641 10.1016/j.cptl.2024.03.008

[14] Miller GE. The assessment of clinical skills/competence/performance. Acad Med. 1990;65(9 Suppl):S63–7. 2400509 10.1097/00001888-199009000-00045

[15] Daniel M, et al. Clinical Reasoning Assessment Methods: A Scoping Review and Practical Guidance. Acad Med. 2019;94(6):902–12. 30720527 10.1097/ACM.0000000000002618

[16] Connor DM, Durning SJ, Rencic JJ. Clinical Reasoning as a Core Competency. Acad Med. 2020;95(8):1166–71. 31577583 10.1097/ACM.0000000000003027

[17] Soledad AR, et al. Using the OSCE to assess medical students’ communication and clinical reasoning during five years of restricted clinical practice. BMC Med Educ. 2025;25(1):608. 40281520 10.1186/s12909-025-07210-x PMC12023525

[18] Siegelman J, et al. Assessment of Clinical Reasoning During a High Stakes Medical Student OSCE. Perspect Med Educ. 2024;13(1):629–34. 39678073 10.5334/pme.1513 PMC11639687

[19] Lubarsky S, et al. Script concordance testing: a review of published validity evidence. Med Educ. 2011;45(4):329–38. 21401680 10.1111/j.1365-2923.2010.03863.x

[20] Régent A, Thampy H, Singh M. Assessing clinical reasoning in the OSCE: pilot-testing a novel oral debrief exercise. BMC Med Educ. 2023;23(1):718. 37789308 10.1186/s12909-023-04668-5 PMC10548592

[21] Vidyarthi AR, et al. Factors associated with medical student clinical reasoning and evidence based medicine practice. Int J Med Educ. 2015;6:142–8. 26547924 10.5116/ijme.563a.5dd0 PMC4646359

[22] Brannick MT, Erol-Korkmaz HT, Prewett M. A systematic review of the reliability of objective structured clinical examination scores. Med Educ. 2011;45(12):1181–9. 21988659 10.1111/j.1365-2923.2011.04075.x

[23] Yazbeck Karam V, et al. Evaluating the validity evidence of an OSCE: results from a new medical school. BMC Med Educ. 2018;18(1):313. 30572876 10.1186/s12909-018-1421-x PMC6302424

[24] Shimizu I, et al. Progress testing of an objective structured clinical examination during undergraduate clinical clerkship: a mixed-methods pilot study. BMC Med Educ. 2023;23(1):958. 38098012 10.1186/s12909-023-04940-8 PMC10720173

[25] Matet A, et al. Impact of integrating objective structured clinical examination into academic student assessment: Large-scale experience in a French medical school. PLoS ONE. 2021;16(1):e0245439. 33444375 10.1371/journal.pone.0245439 PMC7808634

[26] Soffler MI, et al. Raising the Stakes: Assessing Competency with Simulation in Pulmonary and Critical Care Medicine. Ann Am Thorac Soc. 2018;15(9):1024–6. 30011384 10.1513/AnnalsATS.201802-120PS

[27] Park YS, et al. Transition to Residency: National Study of Factors Contributing to Variability in Learner Milestones Ratings in Emergency Medicine and Family Medicine. Acad Med. 2023;98(11s):S123–32. 37983405 10.1097/ACM.0000000000005366

[28] Yeates P, et al. Inter-school variations in the standard of examiners’ graduation-level OSCE judgements. Med Teach. 2025;47(4):735–43. 38976711 10.1080/0142159X.2024.2372087

[29] Rahayu GR, et al. Large-scale multi-site OSCEs for national competency examination of medical doctors in Indonesia. Med Teach. 2016;38(8):801–7. 26380878 10.3109/0142159X.2015.1078890

[30] Wu JW, et al. Comparison of OSCE performance between 6- and 7-year medical school curricula in Taiwan. BMC Med Educ. 2022;22(1):15. 34983486 10.1186/s12909-021-03088-7 PMC8725566

[31] McBee E, et al. Context and clinical reasoning: Understanding the medical student perspective. Perspect Med Educ. 2018;7(4):256–63. 29704167 10.1007/s40037-018-0417-x PMC6086813

[32] Graf J, et al. Communication skills of medical students during the OSCE: Gender-specific differences in a longitudinal trend study. BMC Med Educ. 2017;17(1):75. 28464857 10.1186/s12909-017-0913-4 PMC5414383

[33] Ali A, et al. Gender differences in the acquisition of surgical skills: a systematic review. Surg Endosc. 2015;29(11):3065–73. 25631116 10.1007/s00464-015-4092-2

[34] Klein R, et al. Gender Bias in Resident Assessment in Graduate Medical Education: Review of the Literature. J Gen Intern Med. 2019;34(5):712–9. 30993611 10.1007/s11606-019-04884-0 PMC6502889

[35] Menchetti I, et al. Gender differences in emergency medicine resident assessment: A scoping review. AEM Educ Train. 2022;6(5):e10808. 36189450 10.1002/aet2.10808 PMC9513437

[36] Madrazo L, et al. Self-assessment differences between genders in a low-stakes objective structured clinical examination (OSCE). BMC Res Notes. 2018;11(1):393. 29903050 10.1186/s13104-018-3494-3 PMC6003209

[37] Dwivedi NR, et al. Comparing Standard Setting Methods for Objective Structured Clinical Examinations in a Caribbean Medical School. J Med Educ Curric Dev. 2020;7:2382120520981992. 33447662 10.1177/2382120520981992 PMC7780167

[38] Schoonheim-Klein M, et al. Who will pass the dental OSCE? Comparison of the Angoff and the borderline regression standard setting methods. Eur J Dent Educ. 2009;13(3):162–71. 19630935 10.1111/j.1600-0579.2008.00568.x

